# Facile dione protection to benzo[1,2-*b*:6,5-*b*’]dithiophene-4,5-dione (BDTD) in triggering ultraviolet emission – A new member of the emissive 3,3′-bridged dithiophenes†

**DOI:** 10.1039/d2ra07492c

**Published:** 2023-02-06

**Authors:** Chengpeng Li, Milton J. Kiefel

**Affiliations:** a Institute for Glycomics, Griffith University Parklands Drive Southport QLD 4222 Australia m.kiefel@griffith.edu.au

## Abstract

To date, 3,3′-bridged dithiophenes with bridges developed from the first period elements (either pristine or oxidised) are non emissive. Benzo[1,2-*b*:6,5-*b*']dithiophene-4,5-dione (BDTD) is a typical 3,3′ fused-dithiophene with a dione bridge. It is a critical building block for semiconducting materials, and it is non emissive. We serendipitously discovered that by protecting the diketone of BDTD with ethylene glycol, two isomers (BDTD-5 and 6) were obtained and both compounds effectively emit UV light in solution. Their maximum emission (382 and 375 nm for BDTD-5 and 6, respectively) are independent of the type of solvent. Both compounds exhibited fluorescence intensity enhancement in DMF-H_2_O with the increase of water fraction from 0–90%. BDTD-6 can also effectively emit in its crystalline state with a quantum yield (QY) of 14% and an average fluorescence lifetime of 1.6 ns. X-ray crystallographic analysis indicates that BDTD-6 possesses a 3D C–H^…^O interaction structure which produced its effective emission in the crystalline state. These two isomers not only have enlarged the emissive members of the 3,3′-fused dithiophene family, but also expand the emission boundary of emitters in this category to the UV area.

## Abstract 2

We report the solid-state emissive properties and X-ray crystallographic analysis of some 3,3′-fused dithiophene derivatives.

## Graphical abstract

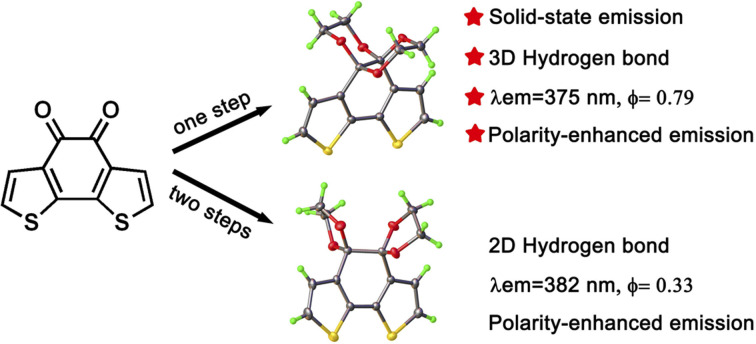

## Introduction

Solid-state emissive organic chromophores have never failed to stimulate scientists to synthesize new compounds with the aim of achieving highly efficient emissive materials due to their promising applications in organic light-emitting diodes (OLED),^1^ organic solid-state lasers,^2,3^ sensors and biological imaging.^4–7^ The frequency at which these emissive materials emit is also of key importance, as exemplified in the recent review by Chen and Xu^8^ discussing electroluminescent materials in the near ultraviolet region (300–400 nm). Several 3,3′-bridged dithiophene compounds are emissive in the solid state, and also due to the large π surface, they have been widely employed as building blocks for constructing photo or electroluminescent materials.^9^ The identity of the bridge largely determines the emission quality, and therefore there has been some interest in the study of the bridges to explore potential high qualified emitters.^10–13^ To date, bridge permutations have covered most chalcogen, pnictogen, and tetrel atoms, as well as the expansion to single, double bonds or even three-atom bridges, but no more than three. Unfortunately, not all of the bridged dithiophenes are emissive, and even the emissive ones have variable emission depending on the size of the bridge, the substituent on the bridge, and the nature of the substituent on the dithiophene rings.^8,9^

**Scheme 1:**
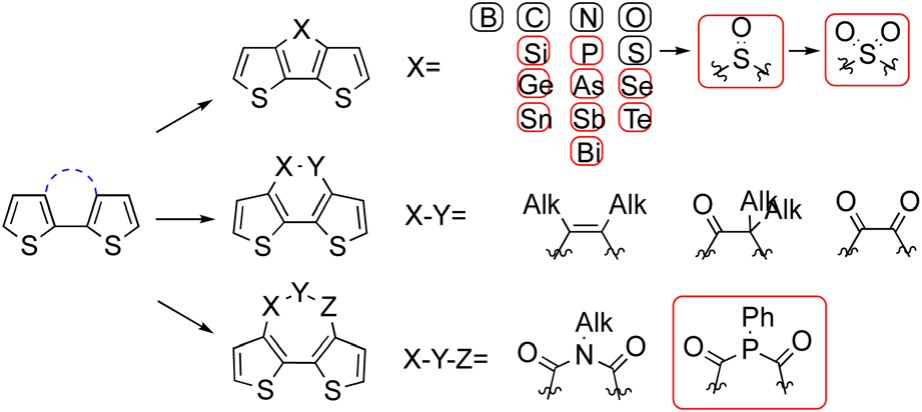
Existing bridges for 3,3′-fused dithiophenes, groups in red are emissive, substitution to dithiophene are omitted for clearance.

As Scheme 1 shows, single-atom fusion in the third positions of dithiophene has been more intensively studied than fusions in other types, possibly because of the less challenging synthesis and longer history of research. To date, bridge atoms from the second period elements are none emissive, no matter if these atoms are in their pristine or oxidized state. In contrast, bridges from the third period start to give emissions except for sulfur, although it was subsequently realised that it is able to emit in its oxidized form.^14,15^ As a second-period member, silicon fused dithiophenes, termed dithieosiloles can emit both in solution and in solid state. The absorption maximum of unsubstituted dithieosilole appears at 345 nm, and the emission maximum is 420 nm.^16,17^ With functional groups attached to the dithiophene framework, both absorption and emission red shifted accordingly.^17^ This is also true for phosphorous-bridged dithiophenes, termed dithieophospholes. The parent unfunctionalized 4-phenyldithienophosphole shows excitation and emission maxima at 338 and 415 nm, respectively. Further oxidation in the phosphorus bridge resulted a bathochromic shift of emission to 453 nm. Through functionalization at the exocyclic P–R group, at the phosphorus atom, or within the main framework, dithienophosphole-based emitters developed thus far have covered the entire visible spectrum.^13^ Unlike silicon and phosphorous, sulfur-fused dithiophenes barely emit light in their pristine state. However, the oxidation of the central sulfur over sulfoxide to sulfone generates bright blue fluorescence. Dithieothiophene-*S*-oxide emits at the maximum of 500 nm, and the emission blue shifts to 450 nm when it is further oxidized to its *S*,*S*-dioxide state.

Although bridges in the fourth period and beyond have been reported, the research around them is limited, and the applications of compounds developed are still in their early stages, possibly because of the synthesis inaccessibility, low quantum yield, or bio-toxicity.^18,19^

Bridge expansion from a single atom to a bond further widens the chemical space around the fused compound. Nonetheless, only carbon–carbon single or double bond have been realized so far, and to the best of our knowledge, the forged dithieobenzene or dithieocyclohexane compounds are all barely emissive.^20–23^ Three-atom bridges mainly focus on the diamide and diketophosphepin component, and the later one reported possessing a faint emission with maximum of 599 nm.^24–26^

More detailed review about the synthesis and photophysical properties of different bridges has been conducted by other groups, and the attention in this research nowadays is often directed more towards material application than towards scaffold modification.^13,27^ For instance, the fluorescence of oxidized DTT was reported two decades ago, where it was established that different substituents resulted in changes to fluorescence.^15^ Subsequently, the most intensely fluorescent derivative has been used to probe antibody binding within cells.^28^ More recently, fluorescent DTT derivatives have been used to probe physical forces within cell membranes.^29,30^

## Results and discussion

### Synthesis of BDTD-5 and BDTD-6

**Scheme 2:**
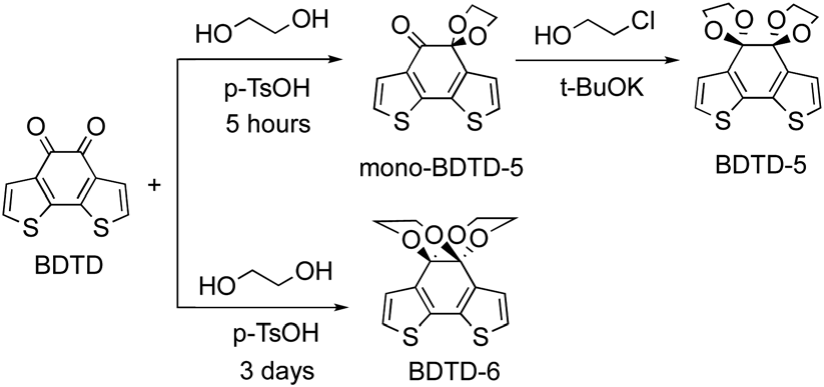
Synthesis of BDTD-5 and BDTD-6.

As part of our exploration of novel fluorescent compounds, we serendipitously discovered that two emissive isomers were produced from a facile ketone protection of benzo[1,2-*b*:6,5-*b*']dithiophene-4,5-dione (BDTD). BDTD itself was synthesised following literature protocols.^23^ Initially attempted diketone protection of BDTD using standard ethylene glycol under acidic catalysis in toluene at reflux afforded a mixture of two products. Careful examination of the NMR spectra suggested the two products were isomers. It was noted that whilst the BDTD-6 isomer could be obtained in good yield (71% under these conditions) the alternative BDTD-5 isomer was only obtained in low yield (<20%). The yield of BDTD-5 could be improved by using a two-step protection sequence, firstly forming the mono-BDTD-5, and then exposure of mono-BDTD-5 to potassium *tert*-butoxide and 2-chloroethanol furnished BDTD-5 in 45% yield (Scheme 2). Our results from the synthesis of BDTD-5 and BDTD-6 are consistent with the observation of other reported analogues.^31^

The BDTD-5 and BDTD-6 isomers have distinctly different NMR spectra (Fig. S1†), but without additional data we could not assign a given NMR spectrum to a particular isomer. Fortunately, both isomers could be crystallised (*vide infra*) to unequivocally match the isomer to the spectra obtained. From the data, it is clear that one isomer forms two distinct 5-membered 1,3-dioxolane rings on each ketone (BDTD-5), whilst the other isomer forms two fused 6-membered 1,4-dioxane rings across two ketones (BDTD-6). As Fig. S1† shows, the ethylene-group hydrogens in BDTD-6 split into two distinctive sets of peaks in the ^1^H NMR spectrum, which can be determined as a set of axial protons and a group of equatorial protons, respectively. The same split can also be found in BDTD-5, although the coupling parameters are much smaller, possibly because its dioxolane rings are not in well-defined “chair” conformation. At the same time, the bridge carbon in BDTD-5 has shifted downfield of 13 ppm compared to that of BDTD-6 (Fig. S2†).

### Photophysical properties and emission of BDTD-5 and BDTD-6

The absorption and fluorescence spectra of both BDTD-5 and BDTD-6 in methanol solution are shown in Fig. S4† and 1, respectively, and the relevant data in various solvents are summarized in Table 1.

**Fig. 1:**
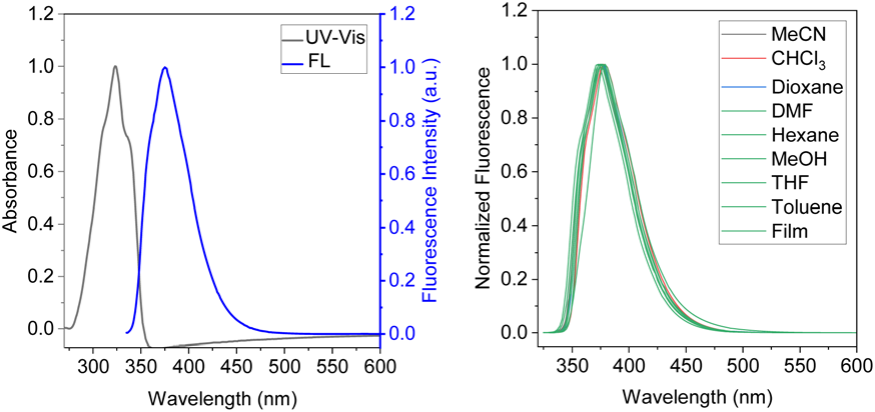
Normalized absorption and emission spectra of BDTD-6 in methanol (5.0 × 10^−6^ mol L^−1^, *λ*_ex_ = 325 nm) (left); normalized fluorescence spectra of BDTD-6 in various solvents and PMMA film, *λ*_ex_ = 325 nm (right).

**Table 1:** Photophysical and emission data of BDTD-5 and BDTD-6

Compound	Condition	*λ*_max_ [nm]^a^	*ε* [M^−1^ c^−1^] (× 10^3^)^b^	*λ*_em_ [nm]^c^	*Φ* d	*τ*_av_/[ns]^e^	*K*_r_/[ns^−1^]^f^	*K*_nr_/[ns^−1^]^g^
BDTD-5	Methanol	328	19.3	382	0.07	0.34	0.93	2.74
	Toluene	329	18.0	384	0.19	0.35	0.81	2.31
	CHCl_3_	331	20.1	387	0.33	0.84	0.67	0.8
	DMF	328	24.4	381	0.05	0.30	0.95	3.18
	Crystal	—	—	ND^h^	ND^h^	ND^h^	—	—
BDTD-6	Methanol	323	10.4	375	0.42	0.99	0.58	0.59
	Toluene	325	12.2	377	0.53	1.43	0.47	0.33
	CHCl_3_	326	14.0	378	0.79	2.20	0.21	0.1
	DMF	325	15.8	379	0.27	0.88	0.73	0.83
	Crystal	—	—	375	0.14	1.60	0.09	0.54

a Absorption wavelength.

b Molar extinction coefficient.

c Fluorescence emission wavelength (excited at 320 nm for BDTD-5 and 326 nm for BDTD-6).

d Fluorescence quantum yield measured with the reference of quinine sulfate, solid-state was determined by the calibrated integrating sphere system.

e Average fluorescence lifetime.

f Radiative rate constant.

g Non-radiative rate constant.

h ND: Fluorescence was too weak to be detected.

The absorption spectra of both compounds in methanol are almost identical, with a distinct single peak in the range of 270–370 nm. The maximum for BDTD-5 and BDTD-6 is located at 328 and 325 nm, respectively. The identical pattern can also be found in their emission spectra concerning a single peak culminated at 382 nm for BDTD-5 and 375 nm for BDTD-6. However, the 5-membered-ring compound emits less efficiently than the 6-membered-ring one, with the quantum yield in methanol for BDTD-5 being 0.07, whilst for BDTD-6 it is 0.42. As Fig. 1 and S5† show, both compounds showed consistent Stokes shift in various solvents and amorphous state. Specifically, the absorbance of BDTD-6 is from 325 nm in various solutions used in this research, and its fluorescence maxima appear at 375–379 nm in these solutions and 379 nm in the PMMA film. Similarly, the fluorescence maxima of BDTD-5 occur at 381–387 nm in various solvents and 384 nm in PMMA film. The nearly unvarying Stokes shifts of both compounds can be ascribed to their rigid structure since they are unlike any other reported donor-accepter fluorophores.^31,32^ Nonetheless, their emission efficiency is astonishingly affected by the variety of solvents (Table 1). Specifically, the fluorescence quantum yield of BDTD-6 in chloroform can get as high as 0.79, while it decreases rapidly to 0.27 in DMF. Similarly, the fluorescence quantum yield of BDTD-5 in chloroform is 0.33 while in DMF it is only 0.05.

**Fig. 2:**
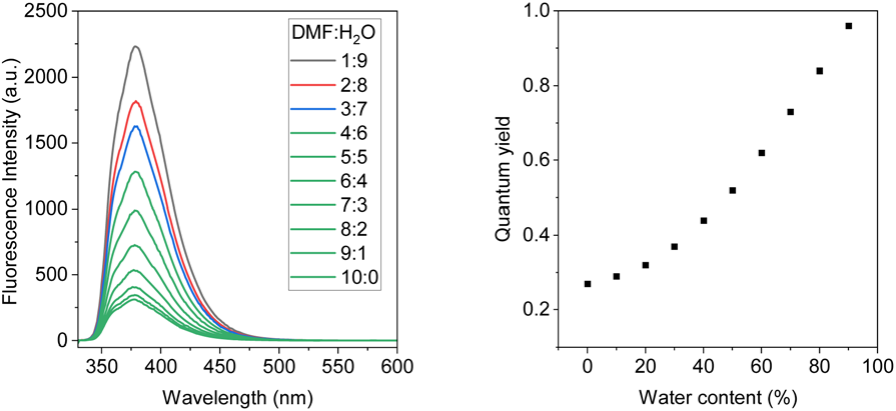
Emission spectra of BDTD-6 in DMF-H_2_O solution with water fraction from 0–90%, 5 × 10^−6^ molL^−1^, *λ*_ex_ = 325 nm (left); the quantum yields of BDTD-6 in varying water concentrations (right).

Further study reveals that the polarity of the solvents significantly affects the emission brightness of both compounds. The fluorescence spectra of both compounds were investigated in DMF-water mixtures with an increase of water fraction. These results are shown in Fig. 2, S6 & S7.† Both compounds exhibited fluorescence intensity enhancement with the increase of water concentration. In pure DMF solution, the quantum yield of BDTD-5 and BDTD-6 is 0.05 and 0.27, respectively. However, when the water fraction increases to 90%, the quantum yield of BDTD-5 gradually increases to 0.56, and BDTD-6 progressively rises to 0.96 (Fig. 2). We believe this observation is consistent with polarity-enhanced emission wherein hydrogen-bonding between the molecules and the solvent enhances emission.^33^ Interestingly, whilst methanol itself has a modest quantum yield for BDTD-5 and BDTD-6, increasing the water concentration also progressively increases the quantum yield (Fig. S8 and S9† for BDTD-6 and S10 and S11 for BDTD-5). It is possible that water can form a hydrogen-bonding bridge between the two cyclic ketal rings thus facilitating the observed polarity-enhanced emission.

As expected, the steady addition of water to the DMF solution causes very limited shifts to the emission maximum of both compounds. Quantum yield determination can be found in the ESI,† and the relevant data for both compounds in DMF-H_2_O solutions are collected in Tables S1 and S2.†

### Single-crystal X-ray structure analysis of BDTD-5 and BDTD-6

For gaining further insight into the characteristic luminescence behaviour between the two BDTD isomers, single-crystal X-ray diffraction (SXRD) has been conducted to analyze relationships between the structures and properties. The crystal and refinement data were summarized in Table S3.† Single crystals of BDTD-5 and BDTD-6 were obtained by slow recrystallization from dichloromethane. BDTD-5 is more likely to grow into tiny crystals, while BDTD-6 can grow into bigger double-terminated ones. Both crystals belong to the same monoclinic system yet crystallize in different space groups.

**Fig. 3:**
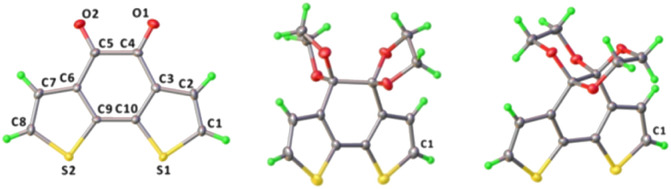
ORTEP diagrams of the molecular structures of BDTD (left), BDTD-5 (middle) and BDTD-6 (right) with thermal ellipsoids shown at 50% probability level.

BDTD-5 crystallizes *P*2_1/_*c* but still with one molecule per asymmetric unit. The unit cell parameters are determined with *a* = 11.394(2) Å, *b* = 8.487(2) Å, *c* = 13.858(3) Å. As Fig. 3 shows, two parallel 1,3-dioxolanes perpendicularly attach to the tricyclic plane, giving rise to a slight twist of the centre ring with the dihedral angle C3–C4–C5–C6 being 51.09°. The connected two thiophene rings also deviate from their original planar skeleton, with the torsion angle C3–C10–C9–C6 being 13.4°. The r.m.s. deviation of non-H tricyclic core atoms from the mean plane in the reported BDTD is 0.02 Å.^34^ However, this value for BDTD-5 is 0.20 Å. Together with r.m.s. deviation value, other selected structure information for comparing BDTD and the two isomers is collected in Table 2.

**Table 2:** Selected structural information for BDTD^a^, BDTD-5 and BDTD-6

Structural information	BDTD^a^	BDTD-5	BDTD-6
Space group	*Pna*2_1_	*P*2_1_/*c*	*Cc*
*a* Å^−1^	12.913	11.394	13.939
*b* Å^−1^	17.070	8.487	12.533
*c* Å^−1^	3.847	13.858	7.407
C3–C4–C5–C6 torsion angle/°	3.90	51.09	39.43
C3–C10–C9–C6 torsion angle/°	0.40	13.40	7.67
r.m.s deviation of tricyclic core Å^−1^	0.02	0.20	0.12
bShortest π⋯π Å^−1^	3.39	7.41	8.49
cMajor atomic contacts/Å	3.44 (S1⋯S2)	2.50 (C8–H⋯O2)	3.55 (S1⋯S2)
			2.56 (C1–H⋯O2)
	3.05 (S2⋯O1)	2.60 (C11–H⋯O1)	2.47 (C8–H⋯O4)
	2.58 (C5–H⋯O1)		2.65 (C2–H⋯O1)
			2.45 (C12–H⋯O1)

a data taken from ref. 34 for comparison.

b Distance between the centroid of two closest parallel tricyclic core.

c The cutoff definition Ar–H⋯O <2.7 Å^35^

In contrast, BDTD-6 crystallizes *Cc* with one molecule per asymmetric unit, and the unit cell parameters are determined with *a* = 13.939(3) Å, *b* = 12.533(2) Å, *c* = 7.407(10) Å. Two 1,4-dioxanes are flanked equally as two wings to the tricyclic plane, which comparatively has less effect on the planarity of the centre ring with the dihedral angle C3–C4–C5–C6 being 39.43° and also less effect to the twist of the two thiophene rings with the torsion angle C3–C10–C9–C6 being 7.67°. As expected, the r.m.s. deviation of the non-H tricyclic core from the mean plane is 0.12 Å which is also smaller than BDTD-5.

**Fig. 4:**
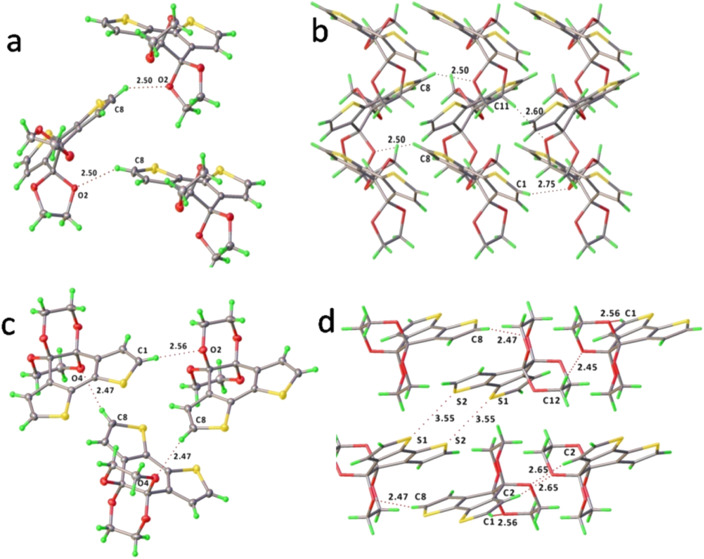
The major intermolecular C–H⋯O interactions and packing motif of BDTD-5 (a and b) and BDTD-6 (c and d).

In comparing the two crystallised structures, it is possible that BDTD-6 emits more efficiently than BDTD-5 because of the higher degree of coplanarity between its two thiophene rings, as well as the more rigidity of its ketal groups. The repeated structural motif in both crystals stacks along their shortest axis, precisely, axis *c* for BDTD-5 and axis *b* for BDTD-6. The bulky cyclic ketals resulting from the ketone protection preclude the formation of the cofacial stacking motif featured in their precursor BDTD. The distance between the centroid of two closest parallel tricyclic cores of BDTD-5 and BDTD-6 is 7.41(1) Å and 8.49(2) Å, respectively. This is larger than that reported for BDTD,^34^ which is 3.39 Å indicating a π⋯π stacking arrangement. Alternatively, the crystal packing in BDTD-5 and BDTD-6 are mainly stabilized by intermolecular C–H⋯O interactions. As Fig. 4 shows, BDTD-5 mainly stacks *via* two sets of C–H⋯O interactions within three molecules. These short contacts occur in the α-carbon hydrogen of thiophene rings and ketal oxygens, thus forming a herringbone arrangement. However, in BDTD-6, three sets of such C–H⋯O intermolecular interactions are formed due to the flip of one of the three molecules, thus resulting in a flattened herringbone pattern. At the same time, intermolecular S⋯S interactions become possible, and an 8-membered ring containing four sulfur atoms forms between two molecules. It is possible that these interactions result in two molecules forming blocks that stabilise the crystal pattern, resulting in BDTD-6 maintaining its capability of emission in the solid state.

**Fig. 5:**
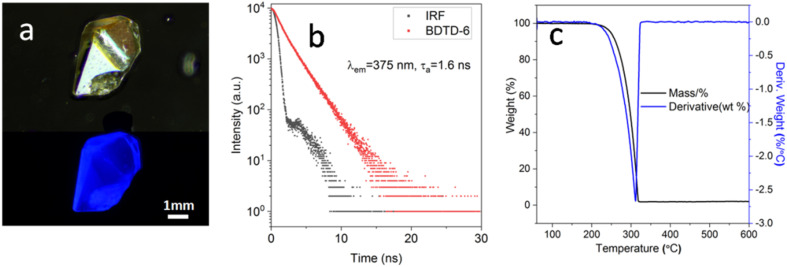
(a) Images of BDTD-6 crystal under daylight (top) and fluorescence (bottom); (b) fluorescence decay profile of BDTD-6 in solid state; (c) TG-DTA curves of BDTD-6.

As mentioned previously, BDTD-6 maintains its emission in the crystalline state as Fig. 5 (a) shows. Further study indicates that the fluorescence quantum yield of BDTD-6 in its crystalline form is 0.14, and the average fluorescence lifetime was calculated to be 1.6 ns (Fig. 5b). Unfortunately, BDTD-5 scarcely emits in its crystalline state, and it is not feasible to get the same data as for BDTD-6.

Considering their potential application in OLED and other optical areas that may require heat treatment,^36,37^ the thermal stability of the isomers was determined by thermogravimetric (TG) and differential thermal analysis (DTA) (Fig. 5c and S10†). Some key parameters are summarized in Table S5.† From the TG curve plot of BDTD-6, this compound was found with no considerable weight loss up to 248 °C and suggested that the solvent in a crystal lattice is absent during the crystallization. The TG curve also describes only one weight-loss stage between 248 °C and 600 °C. At 312 °C in the DTA curve, a sharp thermogram peak corresponds to the BDTD-6 melting point, which correlates well with the TG graph showing maximum weight loss. The sharpness of this peak is indicative of the crystallinity and purity of BDTD-6. BDTD-5 also has similar thermo strength, although its melting point is 23 °C higher than BDTD-6.

### Theoretical DFT calculations

The geometrical and electronic properties of the compounds were carried out with the Gaussian 16 program package, and the single-crystal structure of both compounds was used as the starting structure for geometry optimization.^38^ All calculations were conducted at the B3LYP/6-31G (d) level, and the molecular orbitals were visualized using Gaussview. As shown in Table S4,† the most significant difference in bond length between the DFT optimized structure and single-crystal X-ray diffraction analysis exists in S–C with 0.021 Å, which is very small, confirming the validity of the theoretical level used in this study.

**Fig. 6:**
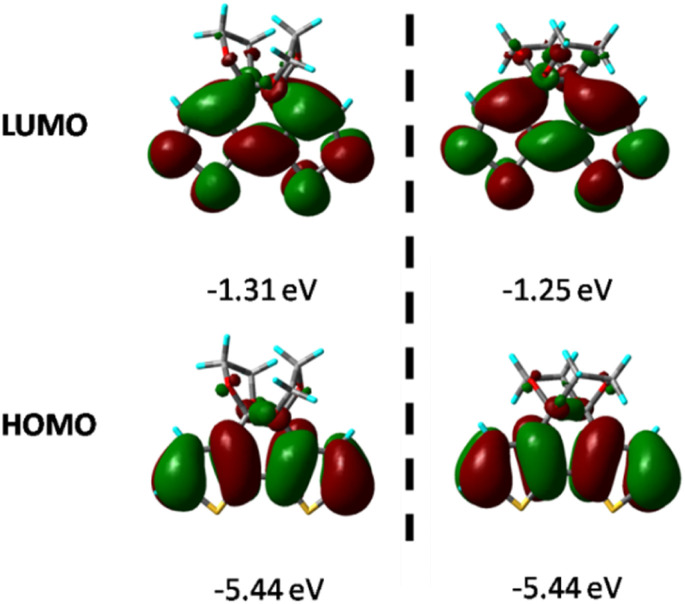
HOMO and LUMO distributions of BDTD-5 (left) and BDTD-6 (right).

Fig. 6 shows the HOMO and LUMO diagrams annotated with the calculated energies. The HOMO of both compounds is just localized over the thiophene rings, and the LUMO covers the whole tricyclic area. Time-dependent DFT calculations further indicate that the optical excitation to S1 is primarily an electronic transition from the HOMO to LUMO, and the energy gap between them for BDTD-5 and 6 are 4.13 and 4.19 eV, respectively, which agrees with the experimental results that the absorption spectrum of BDTD-6 is blue-shifted compared to that of BDTD-5. The calculated energy levels also indicated that the absorption of both compounds mainly involved the π–π* transition from HOMO to LUMO, and the major orbital contribution is 98% and 94% for BDTD-5 and 6, respectively.

**Fig. 7:**
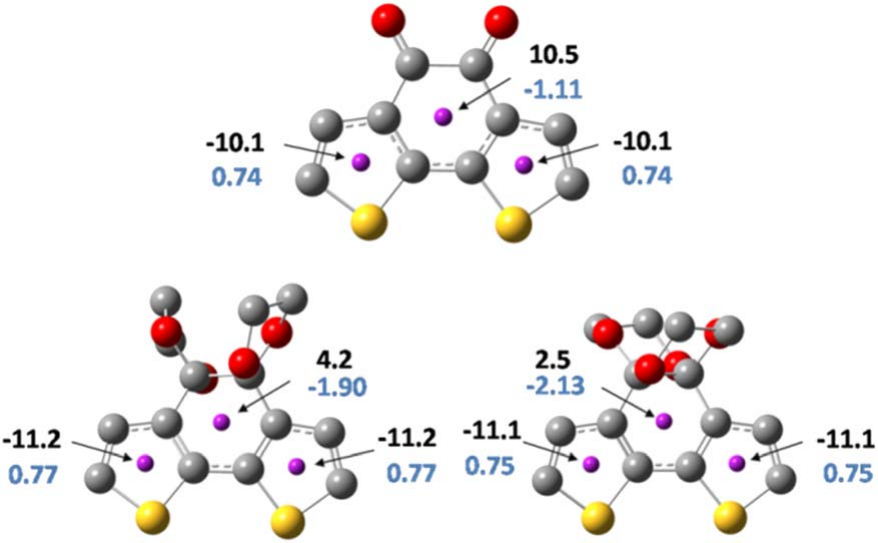
NICS(0)zz values (black), and HOMA values (blue) for BDTD(top), BDTD-5(bottom left) and BDTD-6 (bottom right).

For further understanding of the current density change before and after the protection, the harmonic oscillator measure of aromaticity (HOMA) and Nucleus-independent chemical shift (NICS) was conducted to evaluate the diatropicity or paratropicity properties of the thiophene rings as well as the centre ring of both isomers and the precursor. HOMA was employed as a structural criterion for local aromaticity.^39^ The HOMA values indicate a higher degree of cyclic π-electron delocalization at values close to 1. Fig. 7 shows the HOMA values of the thiophene rings and the core ring in each molecule. As for the thiophene ring, the HOMA values are 0.74, 0.77, and 0.75 of BDTD, BDTD-5 and BDTD-6, respectively, indicating minimal variation through the protection process. At the same time, the HOMA values for the centre ring of these three molecules are negative, which means they are all nonaromatic. Although HOMA is a value for evaluating aromaticity, the proximity of the negative value in each of these three centre rings indicates that the ketone protection scarcely effects the bond length based on which HOMA values are usually calculated.

NICS values provide a convenient and valuable measure for the degree of local ring current effects in π-conjugated systems.^40^ Generally, a ghost atom is placed 1 Å above the ring centre (NICS(1)) to conduct NMR calculations.^41^ By doing this, the effect of the *σ* electrons can be reduced, allowing one to obtain a NICS value that originates mainly from the π electrons. However, due to the 3D conformation of BDTD-5 and BDTD-6 with two oxygen above and under the core ring, the distance between the oxygen and the centre is only 2.8 Å. The ghost atom placed at 1 Å may also be affected by this oxygen. To overcome this, in our calculations the ghost atoms were placed at the centre of each ring (NICS(0)). Taking a step back, the primary purpose of this calculation is to compare the change rather than quantify the values. Consistent with the HOMA values, the NICS values of the core ring in BDTD, BDTD-5 and BDTD-6 are 10.5, 4.2 and 2.5, respectively, which means these ring currents are paratropic. Although this ketalation has dramatically attenuated the antiaromaticity of the core ring, it scarcely affected the thiophene aromaticity. Additionally, the variation of the NICS values between the two isomers is slight and is roughly in proportion to the torsion degree of the centre rings.

## Experimental

### 5*H*-spiro[benzo[1,2-*b*:6,5-*b*']dithiophene-4,2'-[1,3]dioxolan]-5-one (mono-BDTD-5)

Benzo[1,2-*b*:6,5-*b*']dithiophene-4,5-dione (BDTD) (500 mg, 2.27 mmol) and ethylene glycol (1.28 mL, 22.7 mmol) was added to toluene (30 mL) in a round bottom flask equipped with a Dean–Stark. A catalytic amount of *p*-toluenesulfonic acid was added, and the mixture was heated to reflux for 5 hours. TLC showed that most of the starting material has transferred into mono-BDTD-5. At the same time, BDTD-6 was starting to appear. The reaction mixture was allowed to cool to room temperature before the solvent was removed by rotary evaporation under reduced pressure. The crude product was purified by silica gel column chromatography with hexanes/ethyl acetate (5 : 1, v/v) to give mono-BDTD-5 as a grey solid, 348 mg, 60% yield. A small portion of BDTD-6 was also collected from the column (145 mg, 21%). ^1^H NMR (400 MHz, CDCl_3_) *δ* 7.31 (d, *J* = 5.2 Hz, 2H), 7.19 (d, *J* = 5.1 Hz, 2H), 7.08 (d, *J* = 5.1 Hz, 2H), 6.99 (d, *J* = 5.2 Hz, 2H), 4.44–4.41 (m, 2H), 4.26–4.23 (m, 2H); ^13^C NMR (101 MHz, CDCl_3_) *δ* 189.95, 144.30, 137.93, 131.24, 130.51, 126.37, 124.94, 122.36, 65.06; LCMS: *m*/*z*: calcd for C_10_H_4_O_2_S_2_: 220.27; found: 221.1 (M + H)^+^.

### Benzo[1,2-*b*:6,5-*b*']dithiophene-4,5-bis(1,3-dioxolane) (BDTD-5)

5*H*-spiro[benzo[1,2-*b*:6,5-*b*']dithiophene-4,2'-[1,3]dioxolan]-5-one (350 mg, 1.6 mmol), and 2-chloroethanol (0.34 mL, 4.8 mmol) were dissolved in a mixture of anhydrous THF (10 mL) and DMF (30 mL). The resulting mixture was stirred at −78 °C with a dry ice/acetone bath, and then a solution of *t*-BuOK (530 mg, 4.8 mmol) in anhydrous DMF (10 mL) was added dropwise. After stirring at this temperature for 8 hours, the reaction mixture was allowed to warm to room temperature. The mixture was poured into aqueous NH_4_Cl (100 mL, 5%) and extracted with ethyl acetate (10 mL x3). The combined organic phase was washed with brine and then dried with Na_2_SO_4_. The solvent was removed under reduced pressure, and the residue was purified by silica gel column chromatography with hexanes/ethyl acetate (5 : 1, v/v) to give a greenish crystalline product. Yield: 45%. ^1^H NMR (800 MHz, CDCl_3_) *δ* 7.11 (d, *J* = 5.2 Hz, 2H), 7.07 (d, *J* = 5.2 Hz, 2H), 4.29–4.14 (m, 4H); ^13^C NMR (200 MHz, CDCl_3_) *δ* 137.77, 132.56, 125.21, 123.27, 108.28, 66.59; LCMS: *m*/*z*: calcd for C_14_H_12_O_4_S_2_: 308.02; found: 308.9 (M + H)^+^.

### Benzo[1,2-*b*:6,5-*b*']dithiophene-4,5-bis(2,4-dioxane) (BDTD-6)

Benzo[1,2-*b*:6,5-*b*']dithiophene-4,5-dione (BDTD) (500 mg, 2.27 mmol) and ethylene glycol (1.28 mL, 22.7 mmol) was added to toluene (30 mL) in a round bottom flask equipped with a Dean–Stark apparatus. A catalytic amount of *p*-toluenesulfonic acid was added, and the mixture was heated to reflux for one day. Another batch of ethylene glycol (1.28 mL, 22.7 mmol) was added in one portion, and the reaction was allowed to reflux for two more days. TLC showed the starting material and mono-BDTD-5 has completely consumed. The reaction mixture was then cooled to room temperature, and the solvent was removed under reduced pressure. The residue was purified by silica gel column chromatography with hexanes/ethyl acetate (5 : 1, v/v) to give a grey solid, 492 mg, 71% yield.^1^H NMR (800 MHz, CDCl_3_) *δ* 7.20 (d, *J* = 5.2 Hz, 2H), 7.18 (d, *J* = 5.2 Hz, 2H), 4.21–4.14 (m, 2H), 3.75–3.69 (m, 2H); ^13^C NMR (200 MHz, CDCl_3_) *δ* 135.60, 133.42, 125.34, 123.86, 93.79, 61.67; LCMS: *m*/*z*: calcd for C_14_H_12_O_4_S_2_: 308.02; found: 308.9 (M + H)^+^.

## Conclusions

In summary, ketalation of benzo[1,2-*b*:6,5-*b*']dithiophene-4,5-dione resulted in the formation of two isomers, BDTD-5 and BDTD-6, which have been thoroughly and systematically investigated for their emissive properties. This facile ketone protection was mainly accomplished by the conventional ethylene glycol method with a catalytic amount of *p*-toluensulfonic acid. However, BDTD-5 was more efficiently obtained *via* a two-step sequence. Both compounds can efficiently emit UV light in solution, with *λ*_em_ = 382 nm, QY = 7% for BDTD-5 and *λ*_em_ = 375 nm, QY = 42% for BDTD-6 in methanol. Their maximum emissions are scarcely affected by the environment, while the quantum yield and lifetime are significantly influenced by different solvents. Both isomers exhibited polarity enhanced emission when the water fraction increased from 0–90% in a DMF–H_2_O mixture. BDTD-6 can also emit brightly in its crystalline state with the QY = 13%, *τ*_av_ = 1.6 ns. X-ray crystallographic analysis indicated that intermolecular C–H⋯O interactions between thiophene α-carbon hydrogen and ketal oxygen are the dominant force in stabilizing the crystal lattice of both compounds. BDTD-5 was mainly united by two sets of C–H⋯O interactions within three molecules. However, BDTD-6 was united by three sets of such interactions due to the flip of one of those three molecules, making this isomer prone to grow into bigger crystals and emit efficiently in the solid state. TDDFT calculation further indicated that both compounds have the same HOMO whereas different LUMO values, and their emission was mainly generated from π–π* excitation. Both isomers and their precursor were also subjected to HOMA and NICS(1) analysis using DFT methods. The antiaromaticity of the centre ring of these compounds was found to be attenuated by the ketalation.

This research has shed light on triggering solid-state photoluminescence by a simple ketone protection method. The ketone carbon gets saturated without changing its oxidative state, and the π–π stacking between molecules has also been prevented. In addition, the emission discrepancy between BDTD-5 and BDTD-6 explicitly results from the more planar and rigid structure of the later one. This pair of isomers has provided an excellent example in illustrating the significance of molecular coplanarity and rigidity to photoluminescence. Our results show that even relatively small organic molecules can be emissive in the near ultraviolet region, providing a potentially novel and much smaller platform than examples previously reported.^8^ Significantly, this work has enlarged the emissive members of 3,3′-fused-dithiophene family, and for the first time we have shown that the emission can arise from the bridge being made of elements from the second period.

## Author contributions

Chengpeng Li conducted all the experimental work, analysed all the primary spectroscopic, crystallographic, and emissive data, and wrote the first draft of this paper. Milton Kiefel provided overall directional guidance for the research, assisted with interpretation of spectroscopic data, and provided editorial input into the final manuscript.

## Conflicts of interest

There are no conflicts to declare.

## Supplementary Material

RA-013-D2RA07492C-s001

RA-013-D2RA07492C-s002
