# Supplementary material for: Facile dione protection to benzo[1,2-b:6,5-b’]dithiophene-4,5-dione (BDTD) in triggering ultraviolet emission – A new member of the emissive 3,3′-bridged dithiophenes
Source: RSC Adv. 2023 Feb 6;13(7):4713–20. doi: 10.1039/d2ra07492c (PMC9900312; doi:10.1039/d2ra07492c)
Supplement: RA-013-D2RA07492C-s001 [file RA-013-D2RA07492C-s001.pdf]

Electronic Supplementary Material (ESI) for RSC Advances.
This journal is © The Royal Society of Chemistry 2023

Supporting Information

Facile dione protection to benzo[1,2-b:6,5-b']dithiophene-4,5-dione (BDTD) in triggering ultraviolet emission, a new member of emissive 3,3'-bridged dithiophene

Chengpeng Li and Milton Kiefel*

Institute for Glycomics, Griffith University Gold Coast Campus, Southport, Queensland 4222, Australia

Table of content:

Synthesis of precursors 2

NMR spectra for BDTD-5 and 6 4

Fluorescence quantum yield measurement 5

Supporting figures 7

Supporting tables 13

Reference 17

Synthesis of precursors

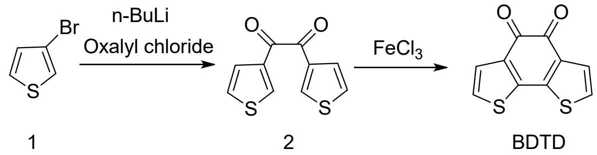

Scheme S1. Synthetic route for BDTD

1,2-di(thiophen-3-yl)ethane-1,2-dione[1]

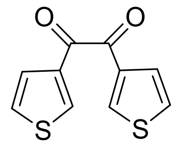

To a solution of n-BuLi (27 mL, 2.5M in hexane) in anhydrous THF (100 mL) 3-bromothiopene (5.7 mL, 61 mmol) was added in drop wise at -78°C. The resulting mixture was allowed to stir for one hour at this temperature and then a solution of LiBr (5.8g, 67 mmol) and CuBr (9.6 g, 67 mmol) in THF (200 mL) was added slowly with an addition funnel maintaining the reaction temperature below -40 °C. After the addition, the reaction mixture was cooled down to -78°C and a solution of oxalyl chloride (2.6 mL, 31 mmol) in THF (10 mL) was added in drop wise. The reaction was kept at -78°C for an hour and then raised to 0°C before quenched with 50 mL of saturated aqueous NH_4Cl. THF was removed by rotary evaporation and 100 mL of ethyl acetate was added to the resulting mixture. The mixture was transferred to a separation funnel and the aqueous phase was extracted with ethyl acetate (20 mL x 2). The combined organic phase was washed with brine before dried with $NaSO_4$. Solvents were then removed by rotary evaporation and the crude product was purified by silica chromatography with hexanes/ethyl acetate (10:1, v/v). A yellow chunky solid was isolated, 5.1g, 74.9% yield. ^{1}H NMR (400 MHz, $CDCl_3$) δ 8.36-8.35 (dd, J = 2.9, 1.2 Hz, 2H), 7.70-7.69 (dd, J = 5.2, 1.2 Hz, 2H), 7.40-7.38 (dd, 5.2, 2.9 Hz, 2H). ^{13}C NMR (101 MHz, $CDCl_3$) δ 185.77, 137.62, 137.41, 127.56,

126.90, LCMS: *m*/*z*: calcd for $C_{14}H_{12}O_4S_2$: 221.98; found: 222.8 $(M + H)^+$.

Benzo[1,2-b:6,5-b']dithiophene-4,5-dione (BDTD)

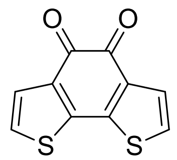

1,2-di(thiophen-3-yl)ethane-1,2-dione (1.8g, 8.1 mmol) was dissolved in dichloromethane (100 mL) and iron trichloride (0.4 g, 24.3 mmol) was then added in one portion. The resulting suspension was allowed to stir over night. 20 mL of water was added to the reaction mixture and stirred for 10 minutes. The resulting solid was filtered and washed with plenty of water. The dark chunk solid was suspended in isopropanol and stirred for over one hour before filtered. The filter cake was then recrystallized from acetonitrile to provide a dark powder, 1.7g, 95.5% yield. ^{1}H NMR (400 MHz, DMSO-d6) δ 7.63-7.62 (d, J = 5.2 Hz, 2H), 7.46-7.45 (d, J = 5.2 Hz, 2H). ^{13}C NMR (101 MHz, DMSO-d6) δ 174.57, 142.93, 135.80, 127.57, 127.55. LCMS: *m*/*z*: calcd for $C_{14}H_{12}O_4S_2$: 219.97; found: 220.9 $(M + H)^+$.

NMR spectra for BDTD-5 and 6

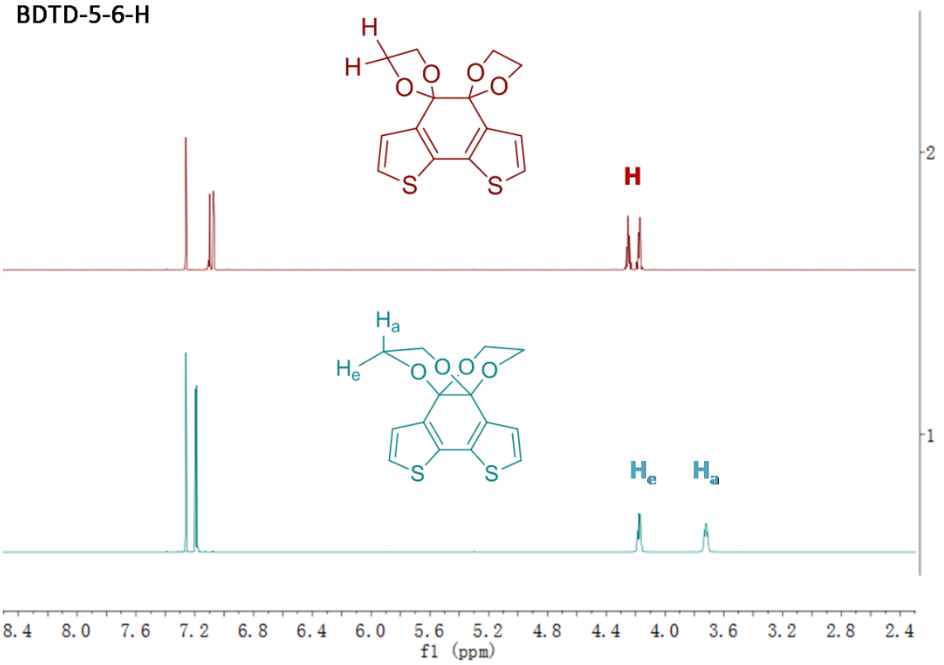

Fig. S1. ^{1}H NMR spectra of BDTD-5 and BDTD-6

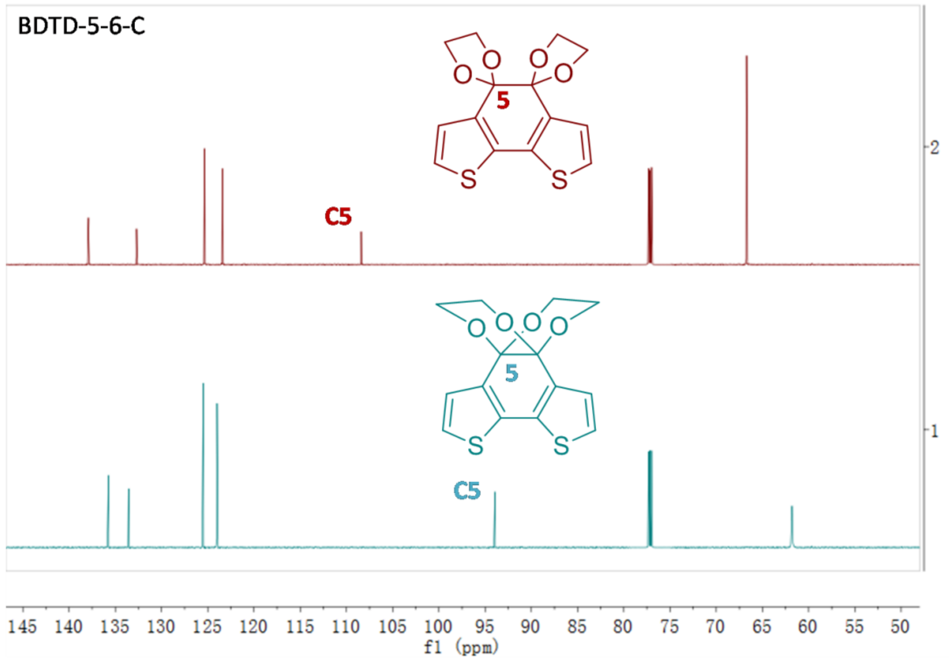

Fig. S2. ^{13}C NMR spectra of BDTD-5 and BDTD-6

Fluorescence quantum yield measurement

The quantum yields of BDTD-5 and BDTD-6 in solutions were quantified by comparing the integrated fluorescence intensity of both samples with the emission of quinine sulfate through the following equation.[2]

$$\emptyset^i = \frac{F^i f_s n_i^2}{F^s f_i n_s^2}\emptyset^s$$

Where $\emptyset^i$ and $\emptyset^s$ are the photoluminescence QY of the sample and the standard, respectively, F^i and F^s are the integrated intensities of sample and standard spectra, f_x is the absorption factor ($f_x = 1 - 10^{-Ax}$, where A = absorbance); the refractive indices of the sample and reference solution are n_i and n_s, respectively.

Quinine sulfate is the most popular standard by far in determining the quantum yield for other compounds, thus we use this reference to record the QY of our two isomers. However, different authors use slightly different QY values based on different literature citations, and the value cited range from 0.51 to 0.60 in different conditions. In this research, we use the QY value of 0.55, and the condition for this value is the quinine sulfate in a solution of sulfuric acid (0.05 mol L^{-1}) with excitation wavelength of 366 nm. Due to the excitation wavelength discrepancy of BDTD isomers and quinine sulfate, we first calibrated the reference value to the according wavelength value of BDTD-5 and 6. Based on the above equation, QY values of quinine sulfate with excitation at 328 nm and 325 nm are calibrated to 0.70 and 0.69, respectively. The QY of BDTD-5 and BDTD-6 are thus calculated based on these two values.

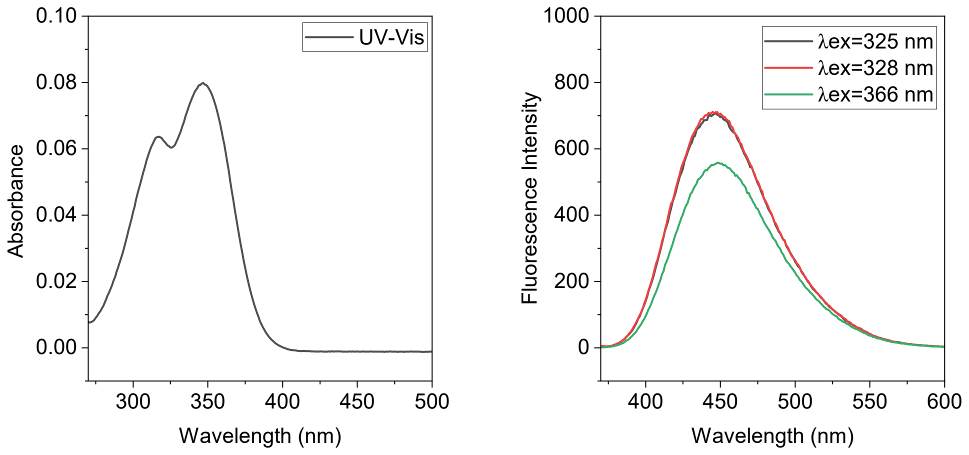

Fig. S3. Absorbance spectrum of quinine sulfate in diluted sulfuric-acid solution (0.05 mol L^{-1}) (left); fluorescence spectra of quinine sulfate at different excitation wavelength (right)

Supporting figures

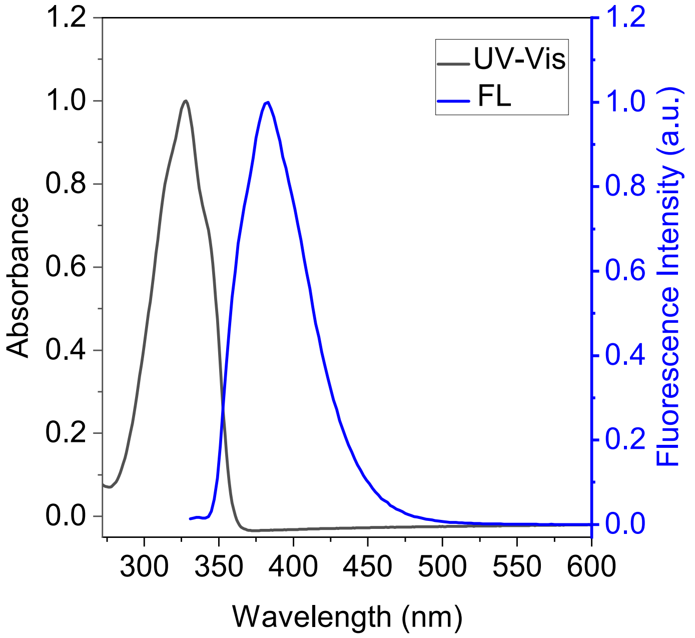

Fig. S4. Normalized absorption and emission spectra of BDTD-5 in methanol (5.0×10^{-6} mol L^{-1}, λ_{ex}=328 nm)

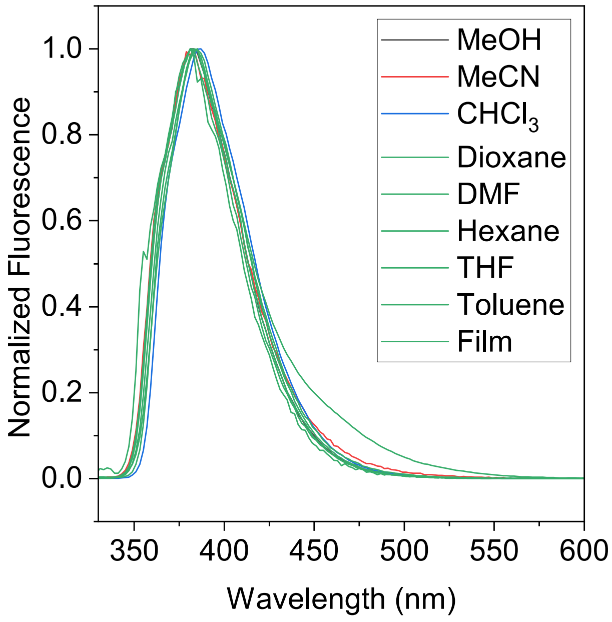

Fig. S5. Normalized fluorescence spectra of BDTD-5 in various solvents and PMMA film (λ_{ex}=328 nm)

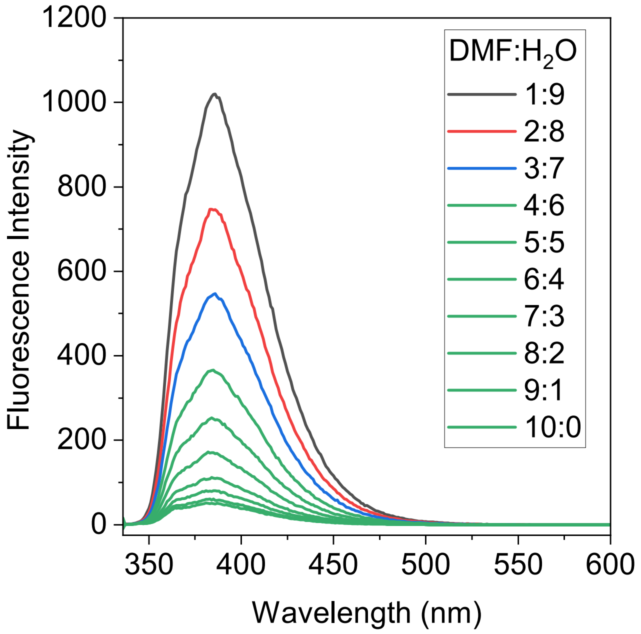

Fig. S6. Emission spectra of BDTD-5 in DMF-H_2O solution with various fractions of H_2O, $5x10^{-6}$ mol L^{-1}, λex=328 nm

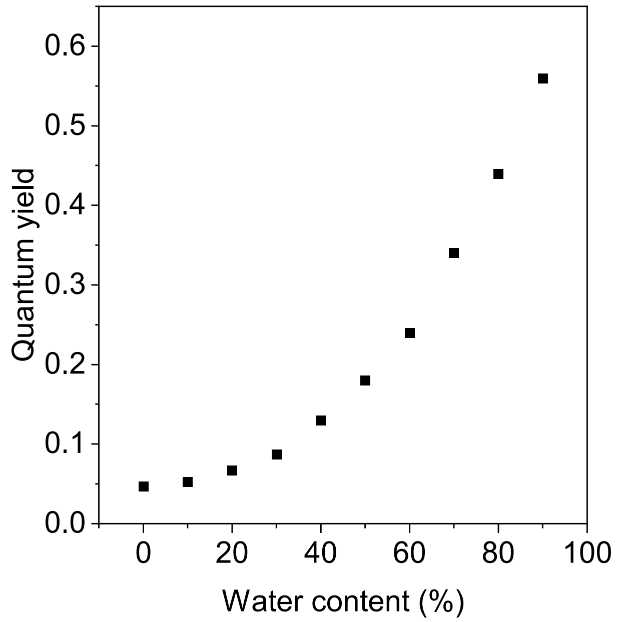

Fig. S7. The quantum yields of BDTD-5 in DMF-H_2O solution with water fraction from 0-90%, $5x10^{-6}$ mol L^{-1}, λex=328 nm

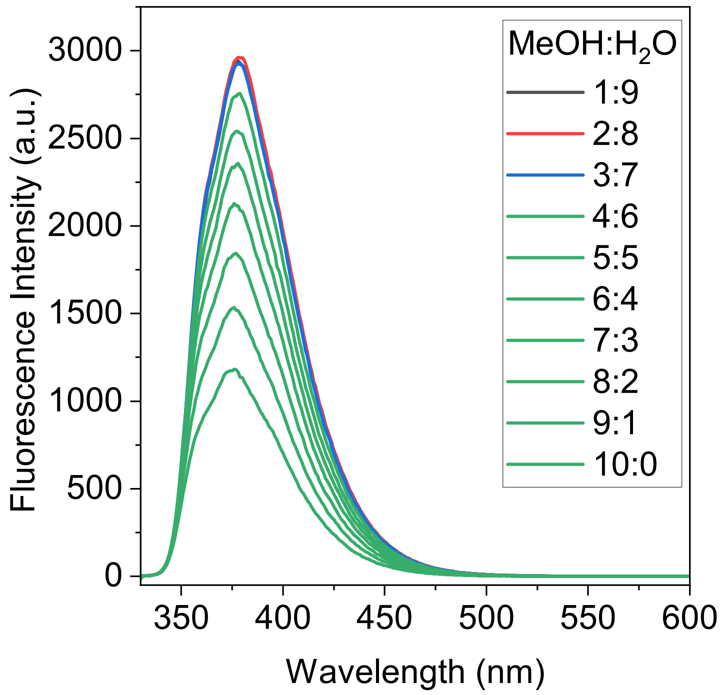

Fig. S8. The emission spectra of BDTD-6 in MeOH-H_2O solution with water fraction from 0-90%, 5x10^{-6} mol L^{-1}, λex=325 nm

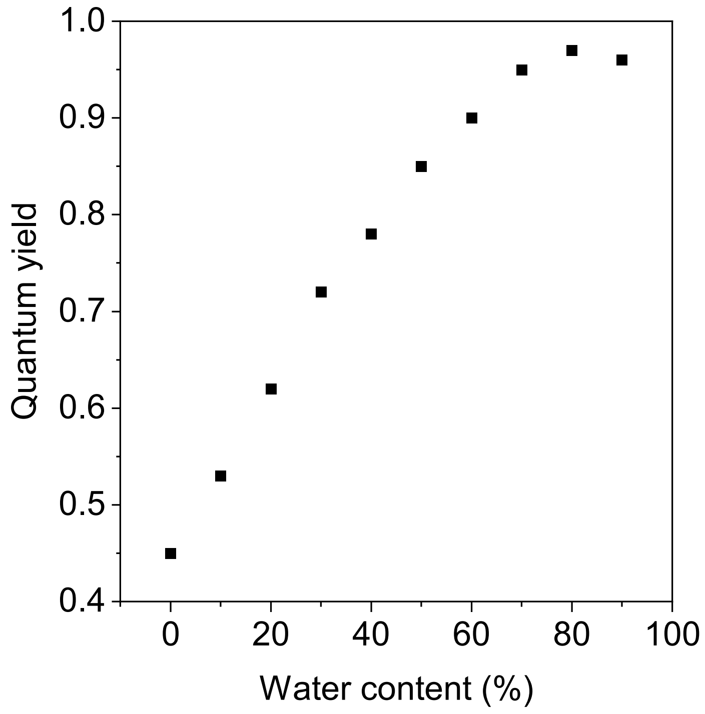

Fig. S9. The quantum yields of BDTD-6 in MeOH-H_2O solution with water fraction from 0-90%, 5x10^{-6} mol L^{-1}, λex=325 nm

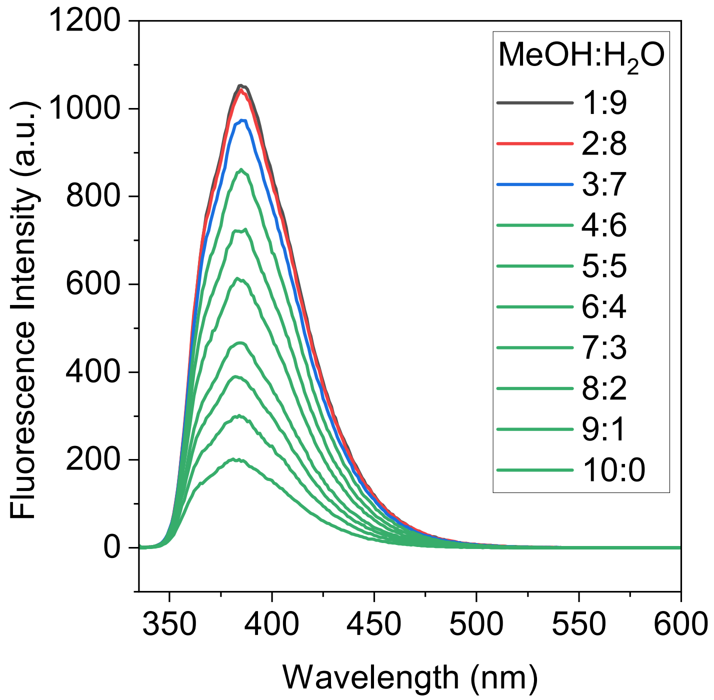

Fig. S10. The emission spectra of BDTD-5 in MeOH-H_2O solution with water fraction from 0-90%, $5x10^{-6}$ mol L^{-1}, λex=328 nm

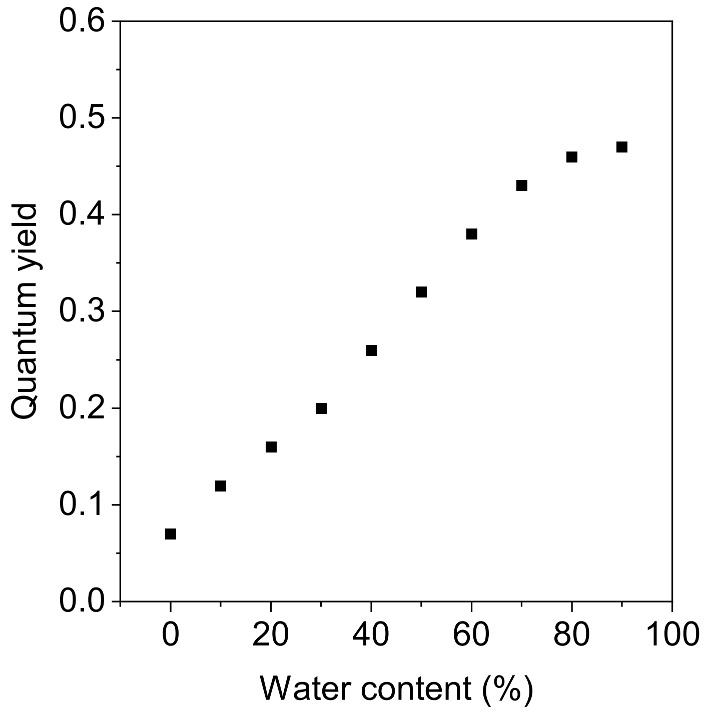

Fig. S11. The quantum yields of BDTD-5 in MeOH-H_2O solution with water fraction from 0-90%, $5x10^{-6}$ mol L^{-1}, λex=328 nm

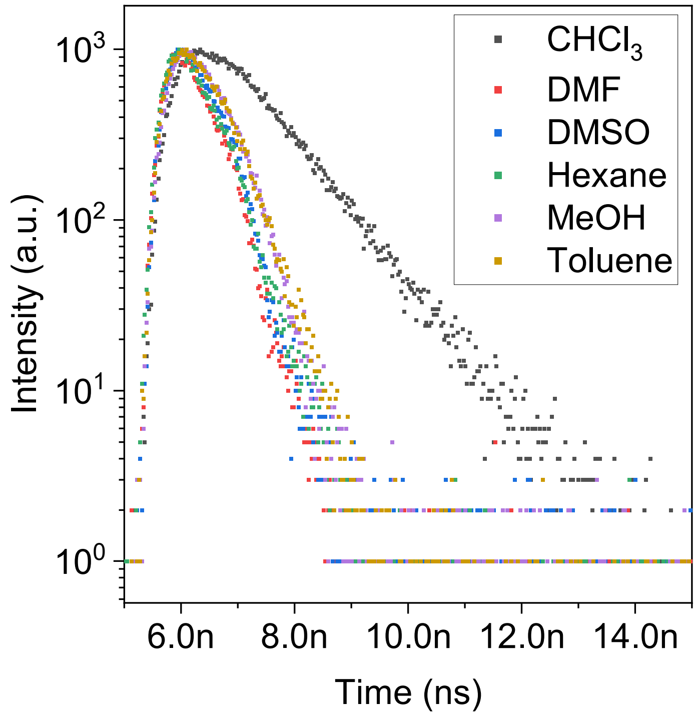

Fig. S12. Photoluminescence lifetime of BDTD-5 in various solvents

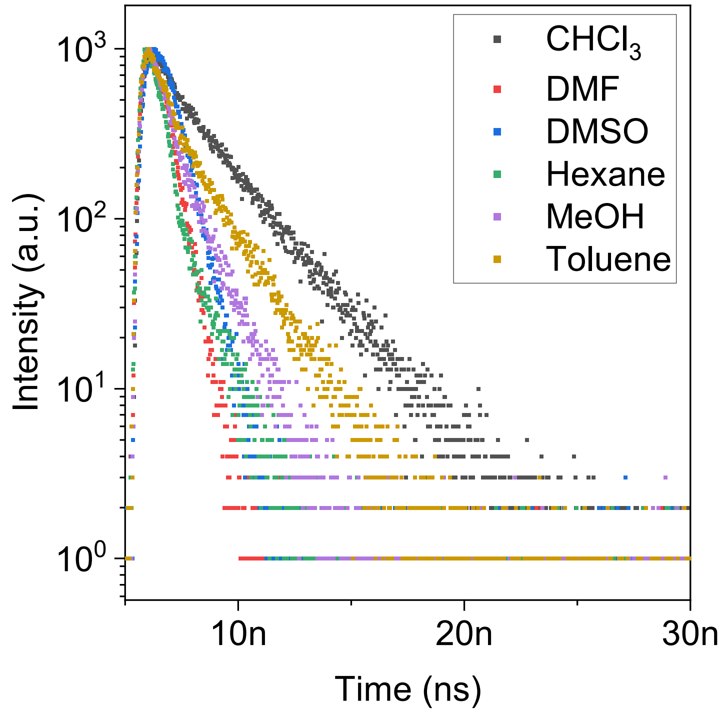

Fig. S13. Photoluminescence lifetime of BDTD-6 in various solvents

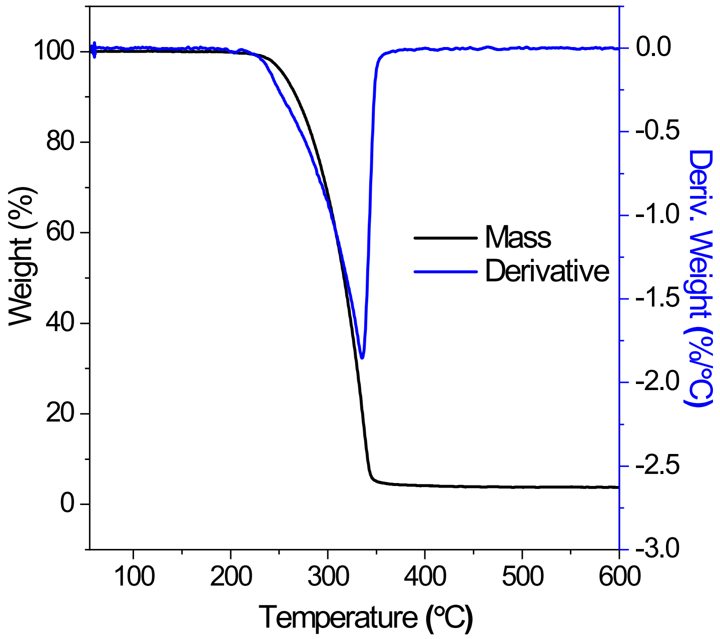

Fig. S14. TG-DTA curves of BDTD-5

Supporting Tables

Table S1. Quantum-yield determination for BDTD-5 in various DMF-H_2O fractions with the reference of quinine sulphate

BDTD-5 DMF:H_2O	Abs	I	Abs(ref)	I(ref)	n(ref)	n	QY(ref)	QY
10:0		2881.715				1.35		0.047
9:1		3385.869				1.315		0.053
8:2		4538.025				1.28		0.067
7:3		6246.1				1.245		0.087
6:4	0.088	9606.635	0.060	57965.68	1.0	1.21	0.70	0.13
5:5		14230.15				1.175		0.18
4:6		20787.91				1.14		0.24
3:7		31180.96				1.105		0.34
2:8		42627.5				1.07		0.44
1:9		58190.68				1.035		0.56

Table S2. Quantum-yield determination for BDTD-6 in various DMF-H_2O fractions with the reference of quinine sulphate

BDTD-6 DMF:H_2O	Abs	I	Abs(ref)	I(ref)	n(ref)	n	QY(ref)	QY
10:0		16017.63				1.35		0.27
9:1		18132.12				1.315		0.29
8:2		21116.99				1.28		0.32
7:3		25808.64				1.245		0.37
6:4	0.079	32492.56	0.060	57965.68	1.0	1.21	0.69	0.44
5:5		40722.05				1.175		0.52
4:6		51580.32				1.14		0.62
3:7		64639.85				1.105		0.73
2:8		79325.67				1.07		0.84
1:9		96893.03				1.035		0.96

Table S3. Crystal data and structure refinement for both BDTD-5 and BDTD-6

Structural information	BDTD-6	BDTD-5
CCDC deposited number	2090190	2090187
Empirical formula	$C_{14}H_{12}O_4S_2$	$C_{14}H_{12}O_4S_2$
Formula weight	308.36	308.36
Temperature/K	293(2)	149.99(10)
Crystal system	monoclinic	monoclinic
Space group	$P2_1/c$	Cc
a/Å	11.3942(2)	13.9393(3)
b/Å	8.4872(2)	12.5328(2)
c/Å	13.8579(3)	7.40660(10)
β/°	103.512(2)	96.018(2)
Volume/Å^3	1303.03(5)	1286.79(4)
Z	4	4
Crystal size/mm^3	n.d.[a]	0.26 × 0.19 × 0.15
Radiation	Cu Kα (λ = 1.54184)	Mo Kα (λ = 0.71073)
2θ range for data collection/°	7.98 to 148.186	6.502 to 73.492
Index ranges	$-13 \le h \le 12$, $-10 \le k \le 10$, $-16 \le l \le 17$	$-23 \le h \le 23$, $-20 \le k \le 20$, $-12 \le l \le 12$
Reflections collected	4951	17191
Independent reflections	2566 [R_{int} = 0.0186, R_{sigma} = 0.0241]	6033 [R_{int} = 0.0269, R_{sigma} = 0.0309]
Data/restraints/parameters	2566/0/181	6033/2/181
Goodness-of-fit on F^2	0.540	1.045
Final R indexes [I>=2σ (I)]	R_1 = 0.0282, wR_2 = 0.0786	R_1 = 0.0287, wR_2 = 0.0732
Final R indexes [all data]	R_1 = 0.0293, wR_2 = 0.0803	R_1 = 0.0302, wR_2 = 0.0748

[a] the crystal dimension was not obtained during crystallographic analysis

Table S4. Bond Lengths of Calculated Geometry and Experimentally Derived Structure of BDTD-5 and 6

BDTD-5	Crystal bond length (Å)	DFT optimized bond length (Å)	Bond length difference (Å)	BDTD-6	Crystal bond length (Å)	DFT optimized bond length (Å)	Bond length difference (Å)
S1-C10	1.7233(14)	1.741	0.018	S2-C9	1.7210(14)	1.741	0.020
S1-C1	1.7223(16)	1.740	0.018	S2-C8	1.7180(16)	1.741	0.023
S2-C9	1.7201(13)	1.741	0.021	S1-C10	1.7245(14)	1.742	0.018
S2-C8	1.7206(18)	1.740	0.021	S1-C1	1.7215(15)	1.741	0.020
O4-C5	1.4259(16)	1.430	0.004	O3-C5	1.4189(16)	1.425	0.006
O4-C14	1.431(2)	1.422	0.009	O3-C13	1.4353(17)	1.429	0.006
O2-C4	1.4262(16)	1.427	0.001	O2-C5	1.4123(16)	1.411	0.001
O2-C12	1.4259(18)	1.426	0.000	O2-C12	1.4455(17)	1.433	0.013
O3-C5	1.4123(17)	1.420	0.008	O4-C4	1.4134(16)	1.411	0.002
O3-C13	1.434(2)	1.432	0.002	O4-C14	1.4420(18)	1.433	0.009
O1-C4	1.4118(16)	1.422	0.010	O1-C4	1.4193(16)	1.425	0.006
O1-C11	1.4315(19)	1.431	0.001	O1-C11	1.4352(18)	1.429	0.006
C10-C3	1.3759(18)	1.379	0.003	C6-C7	1.4190(19)	1.423	0.004
C10-C9	1.4497(19)	1.446	0.004	C6-C9	1.369(2)	1.379	0.010
C3-C4	1.511(2)	1.515	0.004	C6-C5	1.5129(18)	1.516	0.003
C3-C2	1.4159(18)	1.421	0.005	C10-C9	1.4455(19)	1.433	0.013
C4-C5	1.5522(18)	1.566	0.014	C10-C3	1.3725(19)	1.379	0.007
C2-C1	1.368(2)	1.369	0.001	C7-C8	1.363(2)	1.368	0.005
C5-C6	1.5092(19)	1.516	0.007	C4-C5	1.5636(19)	1.58	0.016
C9-C6	1.3751(19)	1.380	0.005	C4-C3	1.5088(19)	1.516	0.007
C6-C7	1.4242(19)	1.422	0.002	C3-C2	1.419(2)	1.423	0.004
C14-C13	1.513(3)	1.524	0.011	C2-C1	1.360(2)	1.368	0.008
C12-C11	1.512(2)	1.526	0.014	C11-C12	1.500(2)	1.514	0.014
C7-C8	1.365(2)	1.369	0.004	C13-C14	1.502(2)	1.514	0.012

Table S5. Comparison of TG data between BDTD-5 and BDTD-6

Compound	T_5[a][°C]	T_{10}[b][°C]	T_{max}[c][°C]
BDTD-5	254	267	335
BDTD-6	248	260	312

a. T_5 is the temperature at 5% weight loss.

b. T_{10} is the temperature at 10% weight loss.

c. Temperature of maximum decomposition rate.

References

1. C. A. R. Frank A. Arroyave, and John R. Reynolds, *Org. Lett.*, 2012, **14**, 4.
2. A. M. Brouwer, *Pure Appl. Chem.,*, 2011, **83**, 2213–2228.
